# Optic Neuropathies: Current and Future Strategies for Optic Nerve Protection and Repair

**DOI:** 10.3390/ijms24086977

**Published:** 2023-04-10

**Authors:** Neil R. Miller, Rong-Kung Tsai

**Affiliations:** 1 Wilmer Eye Institute, Johns Hopkins University School of Medicine, 600 N. Wolfe St., Baltimore, MD 21205, USA; nrmiller@jhmi.edu; 2 Institute of Eye Research, Hualien Tzu Chi Hospital, Buddhist Tzu Chi Medical Foundation, Hualien 970, Taiwan; 3 Institute of Medical Sciences, Tzu Chi University, Hualien 970, Taiwan

Processes that damage the optic nerve, including elevated intraocular pressure, trauma, ischemia, and compression, often cause visual loss for which there is no current treatment. It has long been believed that patients who experience damage to the optic nerve will never regain useful vision because the nerve cannot regenerate or repair itself. This belief is based on three assumptions: (1) a mammalian retinal ganglion cell (RGC) cannot be prevented from dying once its cell body or its axon has been injured; (2) an injured mammalian RGC whose axon has degenerated cannot be induced to extend a new axon; and (3) even if an injured mammalian RGC could be induced to regenerate, the regenerating axon cannot be directed toward its correct target in the central nervous system (CNS) [1]. In fact, accumulating evidence from experimental studies in mammals, including nonhuman primates, shows that, under certain conditions, RGCs can be prevented from dying despite injury to the cell bodies or their axons, injured RGCs whose axons have degenerated can be induced to extend new axons, and regenerating axons can reach their correct targets in the CNS. Several steps are necessary for the successful treatment of optic nerve injuries. First, the death of RGCs that have been (or have the potential to be) damaged must be prevented. Second, living RGCs whose axons have degenerated must be induced to extend new axons toward their targets in the CNS. Finally, a process of synaptic connection and refinement must occur so that appropriate RGCs are connected to the appropriate target in a retinotopic distribution. Prevention of the death of RGCs usually is referred to as neuroprotection, whereas restoration of the optic nerve function after injury is called neurorepair. In this issue, 18 well-respected scientists and their colleagues review or report the results of their original research in the fields of optic nerve protection, repair, or both.

## 1. Reviews

The optic nerve, similar to most pathways in the mature central nervous system, cannot regenerate if injured, and within days, RGCs begin to die. Research over the past two decades has identified several strategies to enable RGCs to regenerate axons the entire length of the optic nerve, in some cases leading to modest reinnervation of di- and mesencephalic visual relay centers. A review by Wong and Benowitz [2] primarily focuses on the role of the innate immune system in improving RGC survival and axon regeneration, and its synergy with manipulations of signal transduction pathways, transcription factors, and cell-extrinsic suppressors of axon growth. Research in this field hopefully will identify clinically effective strategies to improve vision in patients with currently untreatable losses within 5–10 years.

Epigenetic factors are known to influence tissue development, functionality, and their response to pathophysiology. In their review, Ashok et al. [3] focus on different types of epigenetic regulators and their associated molecular apparatus that affect the optic nerve. They emphasize that a comprehensive understanding of epigenetic regulation in optic nerve development and homeostasis should help unravel novel molecular pathways and pave the way to design blueprints for effective therapeutics to address optic nerve protection, repair, and regeneration.

The goal of neuroprotection in optic neuropathies is to prevent loss of RGCs and to preserve their function. The ideal time window for initiating neuroprotective treatments should be the preclinical period at which RGCs start losing their functional integrity before dying. In their review, Porciatti et al. [4] discuss the noninvasive electrophysiological test known as the pattern electroretinogram (PERG) and emphasize that it can assess the ability of RGCs to generate electrical signals under a protracted degenerative process in both clinical conditions and experimental models, which may have both diagnostic and prognostic value and provide the rationale for early treatment. They emphasize that a PERG also can be used to longitudinally monitor the acute and chronic effects of neuroprotective treatments. Finally, they point out that user-friendly versions of the PERG technology are commercially available for both clinical and experimental use.

One of the most interesting therapies for a variety of acute optic neuropathies is erythropoietin (EPO), which has been shown to have neuroprotective properties in extra-hematopoietic tissues, especially the retina. It is postulated that EPO may interact with its heterodimer receptor (EPOR/βcR) to exert its anti-apoptosis, anti-inflammatory, and anti-oxidation effects in preventing RGC death through different intracellular signaling pathways. In their review, Lai et al. [5] summarize the current pre-clinical studies on EPO in treating glaucomatous optic neuropathy, optic neuritis, NAION, and traumatic optic neuropathy. In addition, they explore future strategies of EPO for optic nerve protection and repair, including advances in EPO derivates and EPO deliveries. These strategies hopefully will lead to a new chapter in the treatment of these and other optic neuropathies.

Primary open angle glaucoma (POAG), a chronic optic neuropathy, remains the leading cause of irreversible blindness worldwide. It is driven in part by the pro-fibrotic cytokine transforming growth factor beta (TGF-β) and leads to extracellular matrix remodeling at the lamina cribrosa of the optic nerve head. Despite an array of medical and surgical treatments targeting the only known modifiable risk factor, raised intraocular pressure, many patients still progress and develop significant visual field loss and eventual blindness. The search for alternative treatment strategies targeting the underlying fibrotic transformation in the optic nerve head and trabecular meshwork in glaucoma is ongoing. MicroRNAs are small non-coding RNAs known to regulate post-transcriptional gene expression. Extensive research has been undertaken to uncover the complex role of miRNAs in gene expression and miRNA dysregulation in fibrotic diseases. MiR-29 is a family of miRNAs which are strongly anti-fibrotic in their effects on the TGF-β signaling pathway and the regulation of extracellular matrix production and deposition. In their review, Smyth et al. [6] discuss the anti-fibrotic effects of miR-29 and the role of miR-29 in ocular pathology and in the development of glaucomatous optic neuropathy. A better understanding of the role of miR-29 in POAG may aid in developing diagnostic and therapeutic strategies for patients with this common optic neuropathy.

A reduction in intraocular pressure remains the only proven treatment for POAG, but it does not prevent further neurodegeneration. In their review, Strickland et al. [7] discuss the three major classes of cells in the human optic nerve head (ONH) that provide support for the lamina cribrosa, lamina cribrosa (LC) cells, glial cells, and scleral fibroblasts, all of which are essential in maintaining healthy RGC axons and demonstrate responses to glaucomatous conditions through extracellular matrix remodeling. The authors discuss these responses and emphasize that understanding the major remodeling pathways in the ONH may be key to developing targeted therapies that reduce deleterious remodeling.

Optic neuritis is an inflammatory condition involving the optic nerve and is the most common acute optic neuropathy in young adults. Optic neuritis can be idiopathic or represent an early manifestation of demyelinating diseases, mostly multiple sclerosis (MS), at least in the Western hemisphere. Other causes include antibody-driven optic neuritis associated with neuromyelitis optica spectrum disorder (NMOSD), myelin oligodendrocyte glycoprotein antibody disease (MOGAD), chronic/relapsing inflammatory optic neuropathy (CRION, often a form of MOGAD), sarcoidosis, and a variety of infectious causes such as Lyme disease, Cat Scratch disease, syphilis, and tuberculosis. Appropriate and timely diagnosis is essential to rapidly decide on the appropriate treatment, maximize visual recovery, and minimize recurrences. Saitakis and Chwalisz [8] review the currently available state-of-the-art treatment strategies for many of these forms of optic neuritis, both in the acute phase and in the long term. The authors also discuss emerging therapeutic approaches and novel steps in the direction of achieving remyelination.

As noted in the review by Saitakis and Chwalisz [8], one of the causes of acute optic neuritis, particularly that associated with simultaneous or sequential transverse myelitis, is the aquaporin 4 (AQP4) antibody-driven condition called neuromyelitis optica. Over the past decade, there have been significant advances in the biologic knowledge on NMOSD, which have resulted in the identification of variable disease phenotypes, biomarkers, and complex inflammatory cascades involved in the disease pathogenesis. Ongoing clinical trials are looking at new treatments targeting NMOSD relapses. The review by Huang et al. [9] is intended to provide an update on recent studies regarding issues related to NMOSD, including the pathophysiology of the disease, the potential use of serum and cerebrospinal fluid cytokines as disease biomarkers, the clinical utilization of ocular coherence tomography, and the comparison of different animal models of NMOSD.

Non-arteritic anterior ischemic optic neuropathy (NAION) is the most common cause of sudden optic nerve (ON)-related vision loss in humans. Fortunately, there are several animal models of the condition. In particular, the rodent NAION model (rNAION) closely resembles clinical NAION in its pathophysiological changes and physiological responses and enables analyses of the specific responses to sudden ischemic axonopathy and of the effectiveness of potential treatments. However, there are anatomic and genetic differences between human and rodent optic nerves, and the inducing factors for the human disease and the model are different. These variables can result in marked differences in lesion development between the two species, as well as differences in the possible responses to various treatments. Bernstein et al. [10] discuss these issues as well as some of the species-associated differences that may be related to ischemic lesion severity and responses. These differences may be important when assessing the potential of any treatment that appears beneficial in rNAION to have a similar effect in human NAION.

Leber hereditary optic neuropathy (LHON) is the most common primary mitochondrial DNA disorder. It is characterized by bilateral severe central subacute vision loss due to specific loss of RGCs and their axons. Historically, treatment options have been quite limited, but ongoing clinical trials show promise, with significant advances being made in the testing of free radical scavengers and gene therapy. Spiegel and Sadun [11] summarize the management strategies and rationale of treatments based on current insights from molecular research. Their review includes preventative recommendations for unaffected genetic carriers, current medical and supportive treatments for those affected, and emerging evidence for future potential therapeutics.

## 2. Original Research

The activation of G-protein-coupled receptor 110 (GPR110) has been shown to stimulate neurite extension in developing neurons and after axon injury in adult mice. In the first paper in this issue, Kwon et al. [12] report that intravitreal injection of GPR110 in adult mice after optic nerve crush significantly reduced axon degeneration and improved axon integrity, RGC preservation, and visual function in wild-type but not in gpr110 knockout mice. They suggest that targeting GPR110 may be a viable strategy for functional recovery after optic nerve injury.

Toomey et al. [13] remind us that secondary optic nerve degeneration occurs after primary optic nerve injury. This spread of damage is thought to relate to mechanisms such as oxidative stress, apoptosis, and blood–brain barrier (BBB) dysfunction that, in turn, damage oligodendrocyte precursor cells (OPCs). However, there may be a time period for therapeutic intervention before permanent damage to the BBB and oligodendrocytes render any treatment useless. To address this issue, these investigators performed a partial optic nerve transection in adult rats and assessed BBB dysfunction, oxidative stress, and proliferation in OPCs. They found that even at 1 day post-injury, there was considerable BBB breach and oxidative DNA damage in OPCs, resulting in apoptosis. The investigators thus emphasize the need to consider early oxidative damage to OPCs in therapeutic efforts to limit secondary degeneration following primary optic nerve injury.

Some animal species have the potential for partial or complete regeneration of neural tissue. Sugitani et al. [14] point out that the fish optic nerve can spontaneously regenerate, with visual function being fully restored within 3–4 months after optic nerve injury. However, the regenerative mechanism behind this remains unknown. These investigators focus on the expression of three Yamanaka factors (Oct4, Sox2, and Klf4: OSK), all well-known inducers of induced pluripotent stem (iPS) cells in the zebrafish retina after optic nerve injury. They found that after optic nerve injury, mRNA expression of OSK was rapidly induced in RGCs, that heat shock factor 1 (HSF1) mRNA was most rapidly induced in the RGCs within 30 min, and that activation of OSK mRNA was completely suppressed by the intraocular injection of HSF1 morpholino prior to optic nerve injury. Their results suggest that the sequential activation of HSF1 and OSK might provide an avenue for RGC regeneration with return of the optic nerve function.

Pioglitazone (PGZ) is a drug that selectively stimulates the nuclear receptor peroxisome proliferator-activated receptor gamma (PPAR-γ) and to a lesser extent PPAR-α. It modulates the transcription of the genes involved in the control of glucose and lipid metabolism in various tissues. Sun et al. [15] assessed the protective effect of PGZ on RGCs when given for 4 weeks before photochemically induced NAION (see the paper in this issue by Bernstein et al. [10]) in diabetic and non-diabetic mice. They found that when given for 4 weeks before NAION induction, PGZ confers significant preservation of RGCs in both diabetic and non-diabetic mice assessed 2 weeks after completion of the 4-week treatment. These results suggest that if one could identify a population at high risk for ION (e.g., patients who already had NAION in one eye and who thus have a 15–20% risk of NAION in the fellow eye or patients who experienced post-cataract surgery ION in one eye and who require cataract surgery in the fellow eye), pre-treatment with a drug such as PGZ might reduce RGC damage and thus prevent severe visual loss in the fellow eye if NAION were to occur.

The most common mutation causing LHON is at site 11778 and is transmitted (similar to all mtDNA) to all maternal lineages. However, not everyone harboring the 11778 mutation develops LHON and men are much more often affected than woman. Nuclear modifier genes have been presumed to affect the penetrance of LHON, but conventional genetic methods have failed to clarify these issues. Cheng et al. [16] performed both whole exome sequencing (WES), a technique used to capture all genetic variations, and genome-wide association studies (GWAS), that generally involve targeted genotyping of specific and pre-selected variants using microarrays, to assess seventeen members of five families, all of whom had the 11778 mutation. Seven of these members had LHON, whereas ten were asymptomatic carriers. Using these techniques, the investigators found several mitochondrial genes with a high percentage of variants as well as several candidate nuclear modifier genes. They conclude that both WES and GWAS can provide highly efficient candidate gene screening functions for patients with a molecular genetic component.

Azithromycin is an antibiotic that also has been shown to be neuroprotective in some studies. Zloto et al. [17] assess the neuroprotective potential of intraperitoneal azithromycin after optic nerve crush injury in wild-type mice and in severely immunodeficient NOD scid gamma (NSG) mice. They found reduced apoptosis and improved RGC preservation in both WT and NSG mice, but much more in the WT than in the NSG. Their results suggest that azithromycin acts by immunomodulation. These findings have implications for the development of drugs to preserve RGCs after acute optic neuropathies.

Another drug that has been shown to exhibit RGC protection is granulocyte colony-stimulating factor (GCSF); however, the mechanisms by which this occurs are unclear. To investigate the mechanisms involved in RGC protection by GCSF, Tsai et al. [18] examined the transcriptome profiles of GCSF-treated adult rat retinas using microarray technology after induction of NAION (rNAION, see the paper by Bernstein et al. [10] in this issue) and demonstrated that GCSF modulates a new pathway, TAF9-P53-TRIAP1-CASP3, to control RGC death and survival after optic nerve infarct.

The AQP4 autoantibodies found in most patients with NMOSD are believed to cross the blood–brain barrier, target astrocytes, activate complement, and eventually lead to astrocyte destruction, demyelination, and axonal damage. However, it is still not clear what the primary pathological event is. Zveik et al. [19] hypothesize that the interaction of AQP4-IgG and astrocytes leads to DNA damage and apoptosis. These investigators studied the effects of sera from seropositive NMO patients and healthy controls (HCs) on astrocyte immune gene expression and viability. They found that sera from seropositive NMO patients led to higher expression of apoptosis-related genes and triggered more apoptosis in astrocytes and a higher expression of immunological genes, including BH3-interacting domain death agonist (BID), compared with sera from HCs. Furthermore, NMO sera increased DNA damage and led to a higher expression of immunological genes that interact with BID (TLR4 and NOD-1). Their findings suggest that sera of seropositive NMO patients may cause astrocytic DNA damage and apoptosis and that this may be one of the mechanisms implicated in the primary pathological event in NMO, thus providing new avenues for therapeutic interventions.

Finally, we are grateful to all the invited researchers who contributed to this Special Issue dealing with optic nerve regeneration and repair. Hopefully, these contributions will have both new and lasting impacts on our quest to restore vision to those who suffer from both acute and chronic optic nerve damage.
